# SARS-CoV-2 RNA in Follicular Fluid, Granulosa Cells, and Oocytes of COVID-19 Infected Women Applying for Assisted Reproductive Technology: SARS-CoV-2 Detection in Follicular Fluid

**DOI:** 10.31661/gmj.v11i.2638

**Published:** 2022-12-05

**Authors:** Sina Vakili, Amir Savardashtaki, Mohammad Ebrahim Parsanezhad, Zahra Mosallanezhad, Sedigheh Foruhari, Soudabeh Sabetian, Maryam Davari Zanjani, Mahnaz Banaei, Neda Pirbonyeh, Bahia Namavar Jahromi

**Affiliations:** ^1^ Infertility Research Center, Shiraz University of Medical Sciences, Shiraz, Iran; ^2^ Department of Biotechnology, School of Advanced Medical Sciences and Technologies, Shiraz University of Medical Sciences, Shiraz, Iran; ^3^ Department of Obstetrics and Gynecology, School of Medicine, Shiraz University of Medical Sciences, Shiraz, Iran; ^4^ Stem Cells Technology Research Center, Shiraz University of Medical Sciences, Shiraz, Iran; ^5^ IVF Center, Ghadir Mother and Child Hospital, Shiraz, Iran; ^6^ Department of Microbiology, Burn and Wound Healing Research Center, Shiraz University of Medical Sciences, Shiraz, Iran

**Keywords:** ARS-Cov-2, Follicular Fluid, Oocyte, Granulosa Cell, Reproduction

## Abstract

**Background:**

The Coronavirus disease 2019 (COVID-19) pandemic has raised concerns regarding the application of assisted reproductive technology (ART) in the world. Many ART programs have been delayed or continued with new precautions due to the ambiguity about vertical transmission and pregnancy safety. Regarding the possible risks of SARS-CoV-2 infection on ART and the resultant embryos, this study aimed to investigate the presence of SARS-CoV-2 in follicular fluid, granulosa cells, and oocytes of COVID-19-infected women undergoing ART.

**Materials and Methods:**

COVID-19-positive polymerase chain reaction tests were reported for five women undergoing ART cycles on the day of oocyte retrieval. SARS-CoV-2 tests were performed on oocytes, granulosa cells, and follicular fluid obtained from these COVID-19-infected women.

**Results:**

SARS-CoV-2 RNA was detected only in one follicular fluid sample; however, other follicular fluid samples, granulosa cells, and oocytes were negative regarding viral RNA.

**Conclusion:**

Given the unknown effects of COVID-19 on human reproduction and ART, strict precautions should be taken during the COVID-19 pandemic.

## Introduction

The coronavirus disease-2019 (COVID-19), caused by the severe acute respiratory syndrome coronavirus 2 (SARS-CoV-2) is now spread all over the world [1][2][3]. The disease has major effects on the human life aspects like education, employment, mental and physical health of individuals, and the world’s economy [4][5][6][7]. The main medical societies in the field of reproductive medicine suggested suspending the beginning of reproductive treatments or performing assisted reproductive technologies (ART) with special considerations for ovulation induction, in vitro-fertilization, intrauterine inseminations, oocyte and sperm cryopreservation, as well as fresh/frozen embryo transfers [8][9]. However, ART services must return to operation, especially in certain cases where postponing treatment could be more harmful than proceeding, such as oncological or low ovarian reserve conditions [8][10]. Many studies have shown that viruses can infect the mammalian oocyte and affect the development of the preimplantation embryo [11][12][13]. SARS-CoV-2 can infect several tissues and organs. SARS-CoV-2 infects tissues by using angiotensin-converting enzyme 2 (ACE2) and CD147 or Basigin (BSG) as a receptor on the host cells [14][15]. The mRNA expression of these genes is demonstrated in most of the human female reproductive tract cells [16], endometrium [17], and the human embryo during the early developmental stages [18]. Human oocytes and granulosa cells also express ACE2 and BSG genes, and the corresponding proteins are present on the membrane of these cells [19]. Barragan *et al.* reported that SARS-CoV-2 viral RNA was not detectable in the oocytes analyzed from two women. However, infection with the SARS-CoV-2 cannot be completely ruled out, especially because of the low sample size of the study [20]. Hypothetically, blood flowing through capillaries could be the source of infection in ovarian follicles, follicular cells including granulosa-theca cells, and the oocyte [19]. Hence, this study aimed to investigate the presence of SARS-CoV-2 RNA in follicular fluid, granulosa cells, and oocytes of COVID-19-infected women undergoing ART.

## Materials and Methods

**Figure-1:**
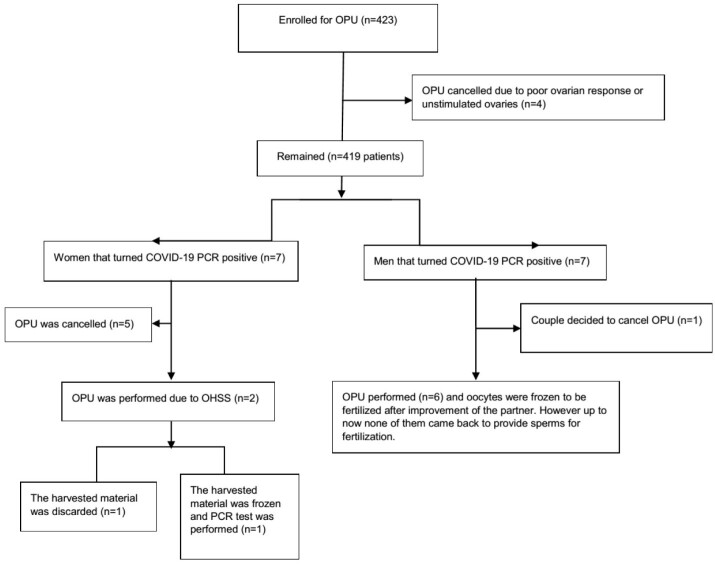
Figure 1. Hazrate zeinab hospital ovum pick-up (OPU) flow chart.

**Figure-2:**
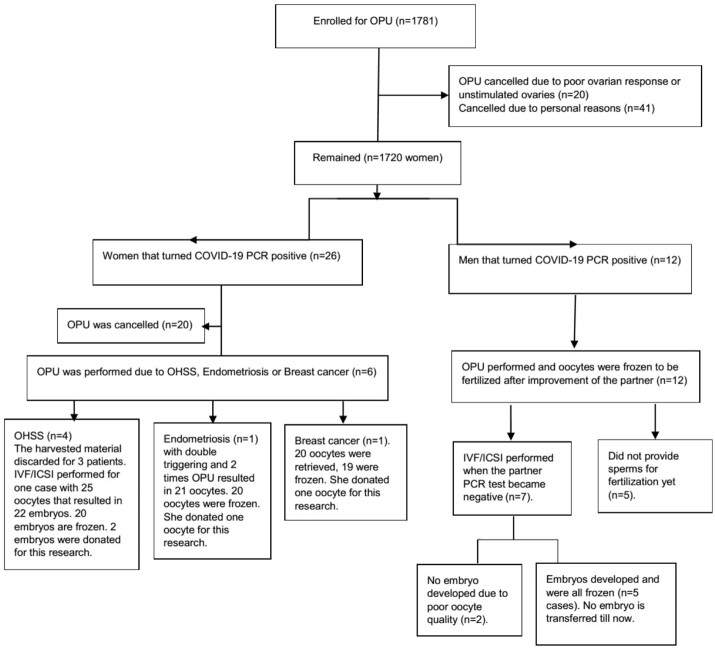
Figure 2. Ghadir Mother and child hospital ovum pick-up (OPU) flow chart.

This cross-sectional study was performed on the harvested oocytes, granulosa cells, and follicular fluid obtained from COVID-19-infected women. According to the instructions of the Ministry of Health and Medical Education (MOHME) of Iran, all the patients who are a candidate for ART during the COVID-19 pandemic should have a negative nasopharyngeal COVID-19 polymerase chain reaction (PCR) test just before starting ovarian stimulation protocols, and also the test should be repeated on the day of final oocyte maturation triggering before oocyte retrieval. In a small number of patients, the SARS-CoV-2 RNA test becomes positive after ovarian stimulation and on the day of oocyte retrieval. Because of great uncertainties regarding the possibility of vertical transmission and the negative effects of the infection on the embryo, treatment is stopped in these patients. However, in a few cases who are prone to develop severe ovarian hyperstimulation syndrome (OHSS) and its related complications, or in some special cases such as endometriosis or breast cancer patients, oocyte retrieval is suggested. According to the current instructions in infertility treatment centers, the obtained follicular fluid and the cells from the OHSS patients are discarded. These unused collected samples from COVID-19-infected women were investigated for the presence of SARS-CoV-2 RNA in their follicular fluid, granulosa cells. Patients were given oral and written information, ample time to consider their participation, and consented in writing before the study. Finally, four SARS-CoV-2 PCR-positive patients entered the study. This study was approved by the Scientific and Ethics Committee of Shiraz University of Medical Sciences (approval code: IR.SUMS.REC.1399.1214).

### Stimulation and Oocyte Collection

A standard GnRH antagonist protocol was carried out for all of the patients. On the second day of the menstrual cycle, after performing a transvaginal ultrasound (TVU) scan, ovarian stimulation was started by Follitropin Alpha (Cinal-F®, CinnaGene, Alborz, Iran) and HMG (PDHoMoG®, Pooyesh Daroo, Tehran, Iran). GnRH antagonist (Cetrotide®, Injection, Powder, 250 μg, Serpero pharmaceutical, Italy) at a dose of 250 μg per day was started on cycle day six or when the leading follicle reached 12 mm. The medications were continued until at least two follicles reached 17-18 mm in diameter. Then 2-3 ampules of GnRH agonist (Variopeptyl®, Injection, 0.1 mg, Varian Pharmed, Tehran, Iran) were injected subcutaneously to trigger the final oocyte maturation. TVU-guided oocyte retrieval was performed about 34-36 hours after triggering. All of the stimulated follicles of patients were retrieved. The obtained follicular fluid was transferred to the embryology laboratory to isolate granulosa cells and oocytes based on a standard isolation protocol. To ensure that there was no blood contamination in the follicular fluid, it was evaluated precisely during the isolation of oocytes and granulosa cells by microscope regarding the presence of blood cells. Figure-1 and -2 show the flow of patients and detailed information about the study participants.

### SARS-CoV-2 RNA Detection Tests for Ovum Pick-Up Candidates

The SARS-CoV-2 test was performed for all women who were candidates for ovum pick-up before starting ovarian stimulation and on the day of triggering. COVID-19 laboratory confirmation was defined as a positive result for SARS-CoV-2 RNA in real-time reverse transcriptase-PCR (RT-PCR) assay on nasopharyngeal swabs. To perform the SARS-CoV-2 test, all patients were referred to laboratories approved by the Shiraz University of Medical Sciences.

### SARS-CoV-2 RNA Detection Tests for the Harvested Samples from the Infected Women

Total RNA was extracted from follicular fluid, scrapped granulosa cells, and oocyte samples using the BehPrep Viral Nucleic Acid Extraction kit (BGene Co, Iran) according to the manufacturer's protocol. The BehPrep Viral Nucleic Acid Extraction is designed and optimized to extract a small amount of viral RNA from cells, bronchoalveolar lavage, nasal and pharyngeal swabs, respiratory discharge, viral transfer media, and body fluids. The oocytes and granulosa cells were isolated from the follicular fluid, the cell-free follicular fluid pooled, and RNA extraction was performed directly on 0.2 ml of it. No concentrating steps or ultracentrifugation was performed. The collected granulosa cells of each subject were pooled, and the RNA was extracted from them and used for real-time PCR. RNA extraction on the oocytes of one subject was performed in a pool; for two subjects, it was performed on a single oocyte (because they donated only one oocyte), and for the other, it was performed on two donated embryos. Real-time PCR was carried out on an Applied Biosystems 7500 System (California, USA) using COVID-19 One-Step RT-PCR Kit (PishtazTeb diagnostics, Tehran, Iran). The primer-probe of this kit adopts the dual-target gene design, which targets the specific conserved sequence encoding the RdRp (RNA-dependent RNA polymerase) region and the nucleocapsid protein N region. The PCR detection system includes an internal control primer probe (RNase P) to avoid false-negative results. The result of internal control provides the sampling and extraction process accuracy. The minimum detection limit of the kit was 200 copies/mL. The interpretation of the results was performed based on the manufacturer's instructions. Real-time PCR was performed in technical triplicates.

## Results

Four women who had positive COVID-19 PCR nasopharyngeal tests on the day of oocyte triggering and were candidates for ovum pick-up as protocol, were included in this study. All the patients were asymptomatic. After specimen collection, the SARS-CoV-2 RNA detection test was performed on the obtained follicular fluid, granulosa cells, oocytes, and four embryos of patients. The results of the internal control verified the accuracy of RNA extraction in the experiments (the Ct of RNase P was lower than 35 and was compatible with the manufacturer’s quality control instruction). Complies with the real-time PCR kit quality control, the Ct of negative controls were not detectable, and the Ct of positive controls was less than 35. SARS-CoV-2 RNA was detected in one follicular fluid sample (Ct less than 40 for both RdRp and N genes in triplicates). The other follicular fluid samples were negative for the virus. Furthermore, SARS-CoV-2 RNA was not detectable in all samples of oocytes and granulosa cells.

## Discussion

To our knowledge, up to now, there is no published study on the detection of SARS-CoV-2 RNA in the follicular fluid from COVID-19 PCR-positive women undergoing ART. It is no evidence whether SARS-CoV-2 can enter and infect the human gametes and how can it possibly affect the development of the human embryo [21].

Results of the present study indicated that SARS-CoV-2 could enter follicular fluid as demonstrated by a positive SARS-CoV-2 RNA test in one of our four follicular fluid samples. However, no viral RNA was detectable in the granulosa cells and oocytes. Since the detection limit of the kit used for viral RNA was 200 copies per ml, the presence of the virus with fewer copy numbers cannot be definitively ruled out in the other tested samples. Data from the literature suggests that viruses such as human hepatitis B virus (HBV) [22][23], herpes simplex virus [24], Hobi-like virus [25], and porcine circovirus type 2 [26][27] can infect the oocyte and affect the development of the pre-implantation embryo. Therefore, the presence of SARS-CoV-2 in the follicular fluid might probably induce negative effects on the oocyte and embryo development.

Data on SARS-CoV-2 presence in human follicular fluid, granulosa cells, and oocytes of infected individuals are poorly available to date. To our knowledge, there is only one published study on the detection of the viral RNA of SARS-CoV-2 in oocytes from women who were positive by PCR, in which the viral RNA for gene N was undetectable in all 16 oocytes tested from two COVID-19-positive women [20]. Nonetheless, no conclusion can be obtained as the number of oocytes analyzed (a total of 16 oocytes) was small and only in two women. Furthermore, the patients were asymptomatic, and it was not possible to determine whether symptomatic women may harbor viral particles in their oocytes. Additionally, the test was performed only on oocytes, and the detection limit of the PCR test was 100 copies per ml in that study [20]. It is hypothesized that even if the virus is not detectable in the oocytes, it might have undesirable effects on the oocytes in other ways, such as altered metabolism, toxins, or changes within the ovarian follicle or ovarian niche [19]. In a study on postmenopausal women with severe COVID-19, all patients were tested for SARS-CoV-2 in vaginal fluid, and all samples were negative for the virus [28].

We agree with the suggested strategy that during the COVID-19 pandemic, even for fertility preservation in cancer patients, it is better to explore follicular fluid and seminal plasma for the virus before cryopreservation [29]. However, it is argued that ART procedures and repeated washings might eliminate the viral load and ART embryo development, and the following ART resulting in pregnancy might be even safer than a natural pregnancy during the COVID-19 pandemic [30].

The present study had some limitations. First, the detection limit of the real-time PCR kit was 200 copies per ml, and lower viral loads might have been missed. Second, the number of patients and samples was small.

## Conclusion

We detected SARS-CoV-2 RNA in one follicular fluid sample out of four. However, the other granulosa cells and oocyte samples were negative regarding viral RNA. Given the possible negative effects of the virus on human reproduction, the presence of SARS-CoV-2 RNA in the follicular fluid mandates strict precautions to be taken with ART. Nevertheless, it is mandatory to perform more extensive studies to confirm the findings of this study and to determine the exact possible effects of SARS-CoV-2 on human reproduction and ART outcomes by following the resulting pregnancies.

## Conflict of Interest

The authors declare that there are no conflicts of interest.
